# Time-resolved mapping in calves reveals bovine herpesvirus 1 shift from mucosal replication to trigeminal ganglion neuroinvasion with promyelocytic leukemia protein-centered host–virus antagonism

**DOI:** 10.1128/spectrum.02698-25

**Published:** 2026-07-23

**Authors:** Yong Wang, Huan Jin, Fanyu Wang, Jing Cheng, Mengyao Cao, Linyi Zhou, Wenxiao Liu, Yongqing Li

**Affiliations:** 1 Institute of Animal Husbandry and Veterinary Medicine, Beijing Academy of Agriculture and Forestry Sciences 107624, Beijing, China; 2 Beijing Key Laboratory for Prevention and Control of Infectious Diseases in Livestock and Poultry, Beijing, China; 3 Sino-UK Joint Laboratory for Prevention & Control of Infectious Diseases in Livestock and Poultry, Beijing, China; 4 School of Medicine, University of Electronic Science and Technology of China, Chengdu, China; 5 Xiangcheng Municipal Bureau of Agriculture and Rural Affairs, Henan Province, China; Wayne State University, Detroit, Michigan, USA

**Keywords:** bovine herpesvirus 1 (BoHV-1), interferon-α (IFN-α), promyelocytic leukemia protein (PML), trigeminal ganglion neuroinvasion, viral antagonism

## Abstract

Although bovine herpesvirus 1 (BoHV-1) causes massive losses of cattle, the transition from mucosal replication to neuroinvasion remains poorly understood. Using a controlled calf model, we integrated quantitative virology and transcriptomics to map its pathogenesis and define the role of promyelocytic leukemia protein (PML). Calves inoculated intranasally and ocularly (1.4 × 10^6^ plaque-forming units/head) were sampled daily (1–14 days post-infection, dpi) for glycoprotein B (gB) qPCR. Tissues were analyzed at 4 and 14 dpi to measure viral DNA via gB-specific qPCR, and for mRNA-seq of trigeminal ganglia (TG). Shedding peaked at 3–6 dpi, being highest in nasal samples, lower in ocular samples, and substantially lower in rectal samples, and declined by 10–14 dpi. At 4 dpi, among the tissues sampled, the tonsils exhibited the highest viral burden. TG exhibited low viral levels at 4 dpi, although they remained detectable at 14 dpi, indicating neuroinvasion. The TG program shifted from early proteostasis priming (4 dpi) to immune/extracellular matrix activation with synaptic repression (14 dpi). In MDBK/Vero cells, IFN-α resulted in higher bovine PML (bPML) levels and enlarged PML nuclear bodies (PML-NBs), reducing very early viral DNA levels, whereas BoHV-1 disrupted PML-NB integrity. The different bPML isoforms exerted different effects on viral infection. STRING analysis revealed a conserved PML–SUMO1–UBE2I–DAXX–SP100 core. These findings delineate the mucosal-to-neuronal trajectory, establish PML as both an effector and viral target in complementary *in vitro* systems, and identify SUMO/ubiquitin-linked proteostasis as a tractable target for antiviral intervention.

**IMPORTANCE:**

Although bovine herpesvirus 1 (BoHV-1) remains a major challenge to cattle health, the early transition from mucosal replication to trigeminal neuroinvasion has not been clearly mapped in natural-host calves. By integrating daily shedding kinetics, tissue viral DNA profiling, and time-resolved trigeminal ganglion transcriptomics, we delineate when and how BoHV-1 reaches the sensory neurons. Promyelocytic leukemia protein (PML) is identified as a key intrinsic antiviral factor that is upregulated by IFN-α and restricts very early viral genome accumulation, while viral BoHV-1-encoded infected cell protein 0 actively dismantles PML nuclear bodies. The discovery of opposing isoform-specific PML functions and a conserved PML–SUMO proteostasis hub provides mechanistic insight into BoHV-1 immune evasion. These findings refine our understanding of the mucosal-to-neuronal trajectory of infection and highlight proteostasis-linked antiviral pathways as promising targets for intervention.

## INTRODUCTION

Bovine herpesvirus 1 (BoHV-1), an alphaherpesvirus and the etiologic agent of infectious bovine rhinotracheitis, causes respiratory, ocular, and reproductive diseases, with substantial economic impacts worldwide (1, 2). After primary replication in the upper-respiratory mucosa, BoHV-1 establishes lifelong latency predominantly in trigeminal ganglion (TG) sensory neurons and can reactivate under stress, driving renewed shedding and transmission (3, 4). Transmission is tightly correlated with mucosal shedding, primarily from the nasal cavity, and ocular shedding has been observed in natural and experimental settings (1). Despite extensive descriptions of the effects of clinical diseases and vaccines on shedding, high-resolution quantitative characterizations that couple early shedding kinetics with parallel tissue distribution in experimentally infected calves remain limited (5, 6).

In addition to requiring innate and adaptive immunity, host control of alphaherpesvirus depends on intrinsic nuclear defenses, primarily via promyelocytic leukemia protein (PML), which assembles PML nuclear bodies (PML-NBs), phase-separated condensates that provide a scaffold for antiviral factors. PML-NB integrity, which requires SUMOylation of PML and other constituents, is enhanced by type I interferons (IFN-α/β), positioning PML at the interface of interferon signaling, proteostasis, and chromatin regulation (5, 7). Alphaherpesviruses have evolved countermeasures against intrinsic antiviral defenses: in herpes simplex virus 1 (HSV-1), the immediate-early ubiquitin ligase infected-cell protein 0 (ICP0) localizes to PML-NBs, promotes ubiquitin-mediated degradation of PML and associated components such as SP100, and disrupts NB architecture to relieve repression of viral gene expression (8–10). BoHV-1 encodes a related RING-finger protein, bovine herpesvirus 1-encoded infected-cell protein 0 (bICP0), which is essential for efficient productive infection and exhibits ICP0-like activities, including disruption of PML-NBs and suppression of IFN-dependent transcription, thereby antagonizing intrinsic and innate antiviral programs (11, 12).

PML exists in multiple isoforms with distinct C-terminal regions that confer isoform-specific antiviral function and NB architecture. This specificity has been explored primarily in human cells and HSV models, and it remains unclear whether the various bovine PML (bPML) isoforms alter BoHV-1 replication differently and how PML-linked proteostasis (SUMO/ubiquitin) and transcriptional networks are reorganized in bovine TGs during early infection (13, 14). Additional layers of pathogenesis involve abundant expression of latency-related (LR) RNA in latently infected sensory neurons, suggesting that LR gene products help govern the latency-reactivation cycle (15, 16). bICP0-mediated inhibition of IFN-dependent transcription is critical for virulence; in mice, BoHV-1 replicates only when type I IFN signaling is ablated (17, 18).

Here, we combined a controlled *in vivo* calf model with quantitative virology and systems-level transcriptomics to define the temporal trajectory of BoHV-1 infection from mucosal replication to TG neuroinvasion. To provide a mechanistic context for these *in vivo* observations, we then used cell culture systems to examine PML-centered intrinsic antiviral responses, focusing on IFN-responsive regulation of bPML, viral disruption of PML-NBs, and isoform-specific effects on very early viral genome accumulation. Together, this integrative approach delineates the progression of BoHV-1 infection in the natural host and frames PML as a candidate host factor and viral target whose functional properties can be mechanistically interrogated in reductionist systems.

## MATERIALS AND METHODS

### Viruses, cells, experimental animals, and overall design

Human embryonic kidney 293T cells and Madin–Darby bovine kidney (MDBK, American Type Culture Collection [ATCC] CCL-22) cells were cultured in DMEM (Gibco/Invitrogen; Thermo Fisher Scientific, Waltham, MA) supplemented with 10% fetal bovine serum (Hyclone, Thermo Fisher Scientific), 1% L-glutamine, and 1% penicillin–streptomycin at 37°C in 5% CO_2_. The cells were passaged with 0.25% trypsin–EDTA and routinely confirmed to be mycoplasma-free.

BoHV-1 (laboratory stock) was propagated in MDBK cells (CCL-22; ATCC, Manassas, VA) maintained in DMEM supplemented with 10% heat-inactivated fetal bovine serum (FBS), and 1% penicillin–streptomycin, at 37°C in 5% CO₂. Vero cells (CCL-81; ATCC) were used for bPML isoform overexpression assays.

Clinically healthy male calves, aged 28–35 days, were acclimatized for ≥7 days and randomly assigned to control or infection groups. Because the calves received colostrum from non-vaccinated dams, circulating maternal antibodies were present at the time of infection. Thus, BoHV-1 replication and host responses in this model reflect infection in the presence of maternally derived immunity, consistent with natural field conditions. Neither the calves nor their dams had any history of BoHV-1 vaccination. Age-matched animals were used to ensure that maternal antibody levels were comparable.

### Inoculation, swab sampling, and tissue processing

Calves in the infection group were inoculated intranasally and ocularly with 1.4 × 10⁶ PFU/head BoHV-1 in sterile PBS. Using sterile polyester swabs, ocular, nasal, and rectal swabs were collected daily from 1 to 14 dpi for qPCR-based shedding analysis, placed into 1 mL of PBS (on ice), vortexed, and stored at −80°C until extraction. Subsets of calves were euthanized at 4 dpi and 14 dpi for tissue collection (palatine tonsil, TG, and upper-airway/lymphoid tissue) and downstream virologic and transcriptomic analyses.

At necropsy (4 or 14 dpi), tissues (tonsil, TG, and other upper respiratory tract [URT]/lymphoid organs) were dissected aseptically, partitioned for DNA/RNA analysis, snap-frozen in liquid nitrogen, and stored at −80°C.

### Virus propagation and titration

BoHV-1 working stocks were generated by infecting near-confluent MDBK monolayers at a multiplicity of infection (MOI) of 1 in serum-free DMEM for 1.5 h, with intermittent rocking. After adsorption, the cells were washed with PBS and incubated in maintenance medium (2% FBS) until the cytopathic effect (CPE) was extensive. The supernatant and cells were freeze-thawed three times, clarified (3,000 *× g*, 10 min), aliquoted, and stored at −80°C. The infectious titer was determined by endpoint dilution using MDBK cells in 96-well plates, and TCID₅₀ values were calculated using the Kärber method.

### DNA extraction and absolute qPCR for BoHV-1 glycoprotein B (gB)

The tubes were vortexed (30 s), incubated at room temperature (1 h), and centrifuged (12,000 rpm for 8 min). For DNA extraction using a DNeasy Blood & Tissue Kit (Qiagen, Hilden, Germany), the supernatant (200 µL) was eluted in 40 µL of nuclease-free water. Tissue (ca. 20 mg–30 mg) was homogenized in ATL buffer using stainless-steel beads (TissueLyser), followed by DNA extraction (DNeasy).

Viral DNA was quantified by absolute qPCR targeting gB using plasmid standards spanning 10⁸–10¹ copies per reaction to construct a standard curve (CFX96, Bio-Rad, Hercules, CA; 20 µL reaction with SYBR Green master mix; 95°C for 2 min; 40 cycles of 95°C for 15 s and 58–60°C for 60 s; melting-curve check). The results are expressed as gB copies per swab or normalized to the amount of input tissue DNA (ng). qPCR of tissue viral DNA and transcript detection were performed using tissue from three calves per group (control, 4, and 14 dpi). No pooling was conducted, and each biological replicate corresponded to an individual calf sample. The primer sequences and efficiencies are listed in Table S1.

### RNA isolation, library construction, and RNA-seq

TG samples (control, 4 dpi, and 14 dpi; *n* = 3 per group) comprising the whole TG, including both neuronal and non-neuronal resident cell populations (satellite glial cells, Schwann cells, and resident immune cells), were homogenized in TRIzol (Invitrogen). Poly(A)^+^ mRNA was enriched with oligo (dT) magnetic beads, reverse-transcribed, and converted to double-stranded cDNA; libraries were prepared according to the manufacturer’s instructions and paired-end sequenced on an Illumina NovaSeq 6000 sequencing system (Illumina, San Diego, CA). The raw reads were quality-filtered with fastp, aligned to the bovine reference genome using HISAT2 and TopHat2, and transcripts were quantified in transcripts per million (TPM) for exploratory QC.

### Differential expression and functional enrichment

Differentially expressed genes (DEGs) were identified using DESeq2 with default normalization and the Wald test. Unless otherwise specified, the thresholds were a false discovery rate (FDR; Benjamini–Hochberg correction) <0.05 and |log₂FC| ≥ 1. Gene Ontology (GO) (Biological Process/Cellular Component/Molecular Function) and Kyoto Encyclopedia of Genes and Genomes (KEGG) over-representation analyses were performed on the DEG sets. Dot plots were used to present gene counts and adjusted *P*-values. EggNOG/COG functional categories were assigned to DEGs to assess category-level dynamics at 4 and 14 dpi.

### Gene set enrichment analysis (GSEA)

Pre-ranked GSEA was performed (using MSigDB/GO collections) by ranking all genes using a signed statistic derived from differential expression analysis (log₂FC × −log₁₀*P*). Enrichment was summarized using a normalized enrichment score (NES) and FDR *q*-value. Leading-edge genes were visualized using Z-score heat maps. The analysis parameters and permutation settings are detailed in the figure legends and Materials and Methods sections.

### Generation of bPML-modified cell lines and isoform expression

#### Plasmids and reagents

The lentiviral expression vector pTK643.EF1α-IRES-GFP, packaging ΔNRF plasmid, and VSV-G plasmid were provided by Prof. Liguo Zhang (Institute of Biophysics, Chinese Academy of Sciences, China). The knockdown backbone pLKO.1-puro was obtained from Addgene. Transfection was performed using Lipofectamine 2000 (Invitrogen), according to the manufacturer’s instructions.

#### Short hairpin RNA (shRNA) design and cloning

shRNAs targeting bovine PML were designed using the siRNA Design resource (Thermo Fisher Scientific) and synthesized by Tsingke (Beijing, China). Annealed, double-stranded oligonucleotides were cloned into pLKO.1-puro using AgeI/EcoRI restriction sites and T4 DNA ligase. The oligo sequences and target coordinates are provided in Table S2. Four independent shRNA constructs targeting bovine PML (shPML-1953, -2041, -2429, and -2496) were evaluated for knockdown efficiency, and the most effective construct was selected to establish stable shPML-MDBK cell lines.

#### Lentivirus production and transduction

Third-generation lentiviruses were produced by co-transfecting HEK293T cells with ΔNRF and VSV-G together with either pLKO.1-bPML shRNA (for knockdown) or pTK643-EF1α-bPML-IRES-GFP (for overexpression). The supernatants were collected at 48 h, clarified, and filtered (0.45 µm). For transduction, 1 × 10^6^ MDBK cells were exposed to 1 mL of lentivirus with 8 µg/mL polybrene and spinoculated at 3,000 rpm for 3 h. The inoculum was replaced with complete medium, and the cells were removed from the medium after 48-72 h for selection or sorting.

#### Generation of stable knockdown and overexpression lines

For PML knockdown, MDBK cells were selected in 4 µg/mL puromycin to establish shPML-MDBK lines, with a non-targeting shRNA as the control. For PML overexpression, MDBK (or 293T) cells transduced with pTK643-EF1α-bPML-IRES-GFP were enriched by FACS (BD Aria III) for GFP^+^ cells to derive PML^Hi^-MDBK (or 293T-bPML) lines. All cell lines were validated for bPML expression and PML-NB features using qRT-PCR, immunoblotting, and immunofluorescence.

For MDBK knockdown or overexpression, stable shPML-MDBK (bPML knockdown) and PML^Hi^-MDBK (bPML overexpression) derivatives were generated by lentiviral transduction (standard third-generation packaging), followed by antibiotic-based selection and validation by qRT-PCR and immunoblotting. Immunofluorescence was used to assess PML-NB abundance and organization.

To examine isoform overexpression in Vero cells, plasmids encoding bPML1–bPML6 (C-terminal isoform diversity) or an empty vector were transfected into Vero cells with lipid-based reagent. Equal amounts of plasmid DNA for each bPML isoform were used during transfection, with the input amount adjusted according to plasmid concentration and volume to ensure comparable expression levels. Expression was verified by immunoblotting; cells were used 24 h post-transfection for infection assays.

While the lentiviral construct expresses a defined bovine PML coding sequence, the anti-bPML antibody detects multiple endogenous or post-translationally modified bPML-immunoreactive species.

### Recombinant bPML expression and purification (prokaryotic system)

Bovine PML cDNA fragments (1,174–1,279 or 1,365–1,800 bp; GenBank OM324027) were synthesized (Tsingke) and cloned into the pET-32a^+^ vector. The constructs were transformed into Transetta (DE3) cells. The cultures were induced using 0.5-1 mM IPTG at 25°C for ca. 7 h. Cells were lysed in 20 mM Tris–HCl (pH 8.0) containing 150 mM NaCl; the clarified lysates were applied to Ni-NTA resin (Thermo Fisher Scientific) and eluted as per the manufacturer’s instructions. The fractions were analyzed via SDS–PAGE and Coomassie staining, and purity was assessed prior to downstream immunization or validation.

### Antibody production and validation

New Zealand White rabbits and BALB/c mice were immunized with purified recombinant bPML according to standard schedules (prime + multiple boosts with adjuvants). For monoclonal antibody generation, splenocytes from immunized BALB/c mice were fused with SP2/0 myeloma cells, and hybridomas were screened by ELISA/immunofluorescence/Western blotting for bPML specificity, yielding clone bPML-2G5. Transient transfection of 293T cells with pCMV-HA-C-bPML was followed by immunofluorescence (using bPML-2G5 primary and appropriate secondary antibodies, with DAPI nuclear counterstaining) and immunoblotting of whole-cell lysates (Table S3).

### IFN-α stimulation, immunoblotting, and immunofluorescence

MDBK cells were treated with recombinant IFN-α (doses and durations are given in the figure legends), lysed in RIPA buffer with protease/phosphatase inhibitors, and probed by immunoblotting with anti-bPML mAb (bPML-2G5) and loading controls. For cell IF, cells on coverslips were fixed in paraformaldehyde, permeabilized with 0.1% Triton X-100, blocked, and incubated with anti-bPML and appropriate secondary antibodies. Nuclei were counterstained with DAPI. For tissue IF, TG sections (4 and 14 dpi) were subjected to antigen retrieval, blocking, and double immunofluorescence with anti-bPML and anti-ICP0 antibodies, followed by confocal imaging under matched acquisition settings.

### Early infection kinetics assay (gB qPCR)

MDBK derivatives (shPML, parental, and PML^Hi^) and Vero cells expressing individual bPML isoforms were infected with BoHV-1 at MOI = 1. At 0.5, 1, 2, 4, 6, and 12 h post-infection (hpi), total DNA was extracted and viral gB copies were quantified via qPCR to assess very early genome accumulation. The results were normalized to the cell input amount and analyzed across time points.

### Disruption of PML-NBs by BoHV-1

To examine viral counteraction of PML restriction, MDBK cells were infected at MOI = 1 and fixed at the onset of CPE for double immunofluorescence (bPML/ICP0). In parallel, TG sections from calves at 4 and 14 dpi were analyzed using double immunofluorescence to visualize PML-NB integrity and subcellular distribution. The image acquisition parameters and antibody details are presented in the figures.

### Protein–protein docking

The bPML–bICP0 interface was modeled using HADDOCK with default restraints and explicit solvent refinement. Clusters were ranked using the HADDOCK score, and interfacial properties (e.g., buried surface area) were computed for the top cluster. The parameterization and evaluation criteria are provided in the figure legends and Materials and Methods section.

### STRING protein–protein interaction (PPI) networks

STRING 11.x was used to construct PML-centered PPI networks, with PML used as the seed protein. First-shell interacting proteins were retrieved for *Homo sapiens* and *Bos taurus* using identical high-confidence parameters (combined score ≥0.7). The resulting networks were based on curated and predicted protein–protein associations within the STRING database, incorporating evidence from curated databases, experimental data, co-expression, and text mining.

Ortholog mapping was conducted using the built-in Ensembl identifiers provided by STRING (based on the corresponding Ensembl release integrated into version 11.x) to ensure cross-species comparability between the human and bovine networks. The canonical PML–SUMO1–UBE2I–DAXX–SP100 core module and species-selective interacting nodes were examined, with edge thickness reflecting the combined interaction scores. Enrichment analyses were performed using the default STRING settings, with correction for multiple testing.

RNA-seq expression values were not used as direct inputs for network construction, but were instead interpreted in the context of these reference PML-centered interaction networks.

### Quantitative RT-PCR

Total RNA was extracted with TRIzol; 1 µg of RNA was reverse-transcribed (using a mix of random hexamers and oligo-dT primers), and qRT-PCR was performed using SYBR chemistry (CFX96) and gene-specific primers (Table S1). Expression was normalized to that of GAPDH using the 2⁻^ΔΔCt^ method. Primer specificity was confirmed using melting-curve analysis.

### Statistical analysis and reproducibility

Unless specified otherwise, data are presented as the mean ± SD from ≥3 independent experiments or animals. Two-way ANOVA with *post hoc* multiple-comparison correction was used for time-course assays and unpaired *t*-tests (or Mann–Whitney tests) for two-group comparisons. Significance in RNA-seq analyses was based on FDR-adjusted *q*-values (with Benjamini–Hochberg correction), whereas for qPCR, immunoblotting, and virology assays, it was based on *P*-values from two-tailed Student’s *t*-tests. The significance threshold was set at *P* < 0.05 (two-sided). Analyses were performed using GraphPad Prism and R 4.x, with defined seeds for stochastic procedures.

## RESULTS

### Replication dynamics and early tissue distribution of BoHV-1 in calves

**Fig 1:**
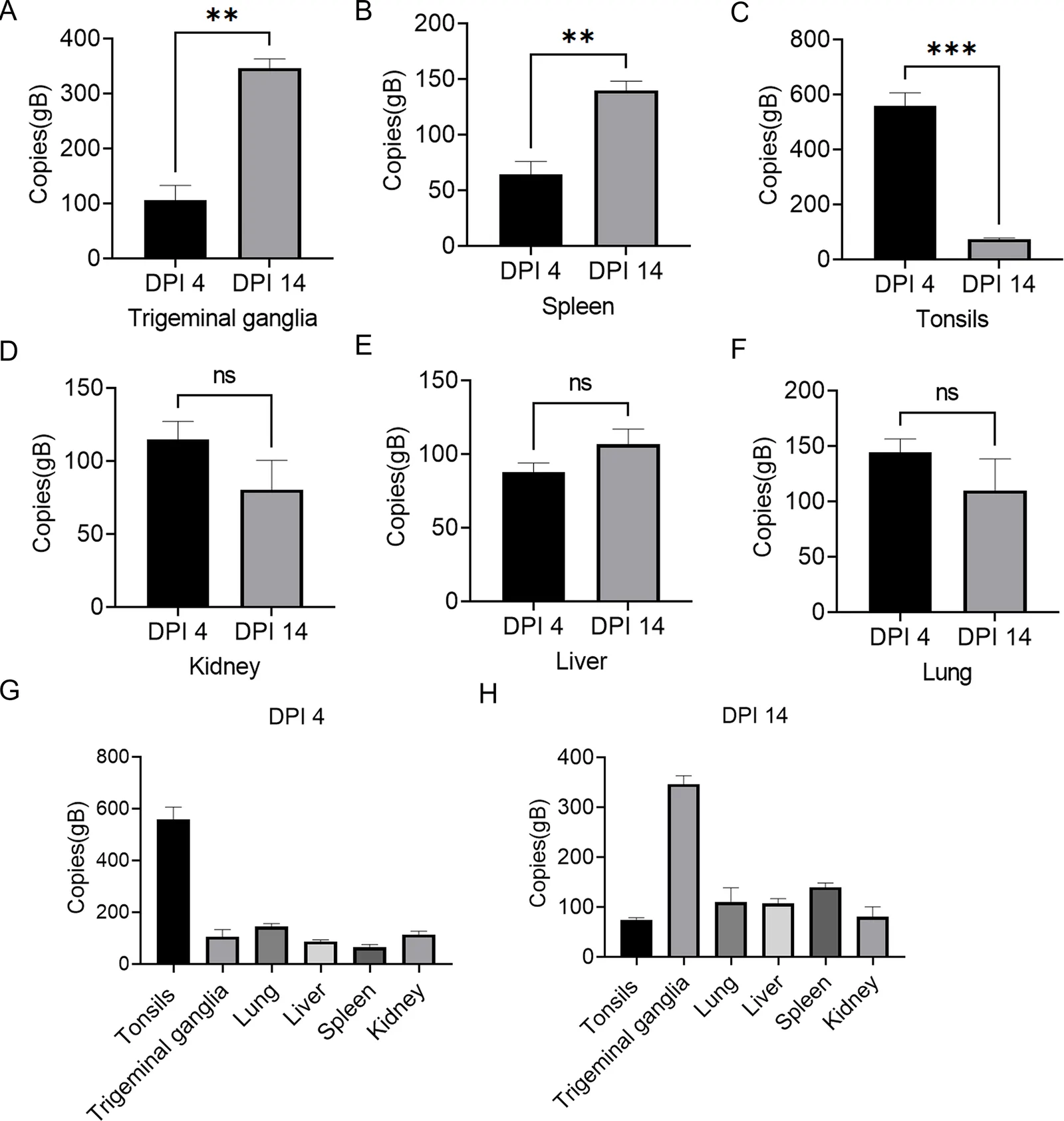
Early distribution of BoHV-1 DNA in the tissue of experimentally infected calves. Calves were inoculated intranasally and ocularly with BoHV-1 (1.4 × 10⁶ PFU per head) and euthanized at 4 or 14 days post-infection (dpi).Trigeminal ganglion (**A**), spleen (**B**), tonsil (**C**), kidney (**D**), liver (**E**), and lung (**F**) were collected. Total DNA was extracted, and viral genomes were quantified by absolute qPCR targeting the gB gene. Bars show gB copy numbers normalized to the amount of input tissue DNA for each animal; horizontal lines denote the group mean ± SD (*n* = 3 calves per time point). (**G**) BoHV-1 genomic DNA levels in individual tissue types collected—palatine tonsil, nasal turbinate, trachea, lung, TG, and the indicated lymphoid tissue—at 4 dpi. Viral DNA was quantified using gB-specific qPCR from whole tissue DNA extracts. (**H**) BoHV-1 genomic DNA levels in the same tissues at 14 dpi, quantified as in panel **G**. Statistical methods and significance criteria are described in the Materials and Methods section. ns, not significant; *P < 0.05; **P < 0.01; ***P < 0.001; ****P < 0.0001.

Mucosal replication was assessed using serial swabbing. At 2 dpi (Fig. S1A and B), viral genomic DNA was detectable in the ocular and rectal swabs. At 3 dpi (Fig. S1C), the nasal swabs exhibited a sharp increase in viral copy number, indicating robust replication and shedding through the upper respiratory tract. To define early tissue distribution, viral DNA was quantified using gB-specific qPCR from whole-tissue DNA extracts obtained from individual tissues (palatine tonsils, nasal turbinate, trachea, lung, trigeminal ganglion, and additional lymphoid tissue) collected at 4 and 14 dpi (Fig. 1A through F). At 4 dpi, BoHV-1 DNA was readily detectable in these tissue types, with the highest copy numbers observed in the palatine tonsils and lower levels in TGs and other tissue types (Fig. 1G). By 14 dpi, viral DNA levels had declined in the palatine tonsils relative to those at 4 dpi, whereas they remained consistently elevated in the TG, indicating neuroinvasion and persistence of viral DNA in the sensory ganglia during the late acute phase (Fig. 1H). Several other tissue types were positive for the viral DNA at both time points, with increased between-tissue heterogeneity observed at 14 dpi. Overall, these data support a progression from robust replication in the upper respiratory and lymphoid tissues to subsequent transport to the TG, with early viral DNA levels persisting into the late acute phase.

### Time-stamped TG transcriptomes reveal early sensing signature and late immune/ECM remodeling during BoHV-1 infection

**Fig 2:**
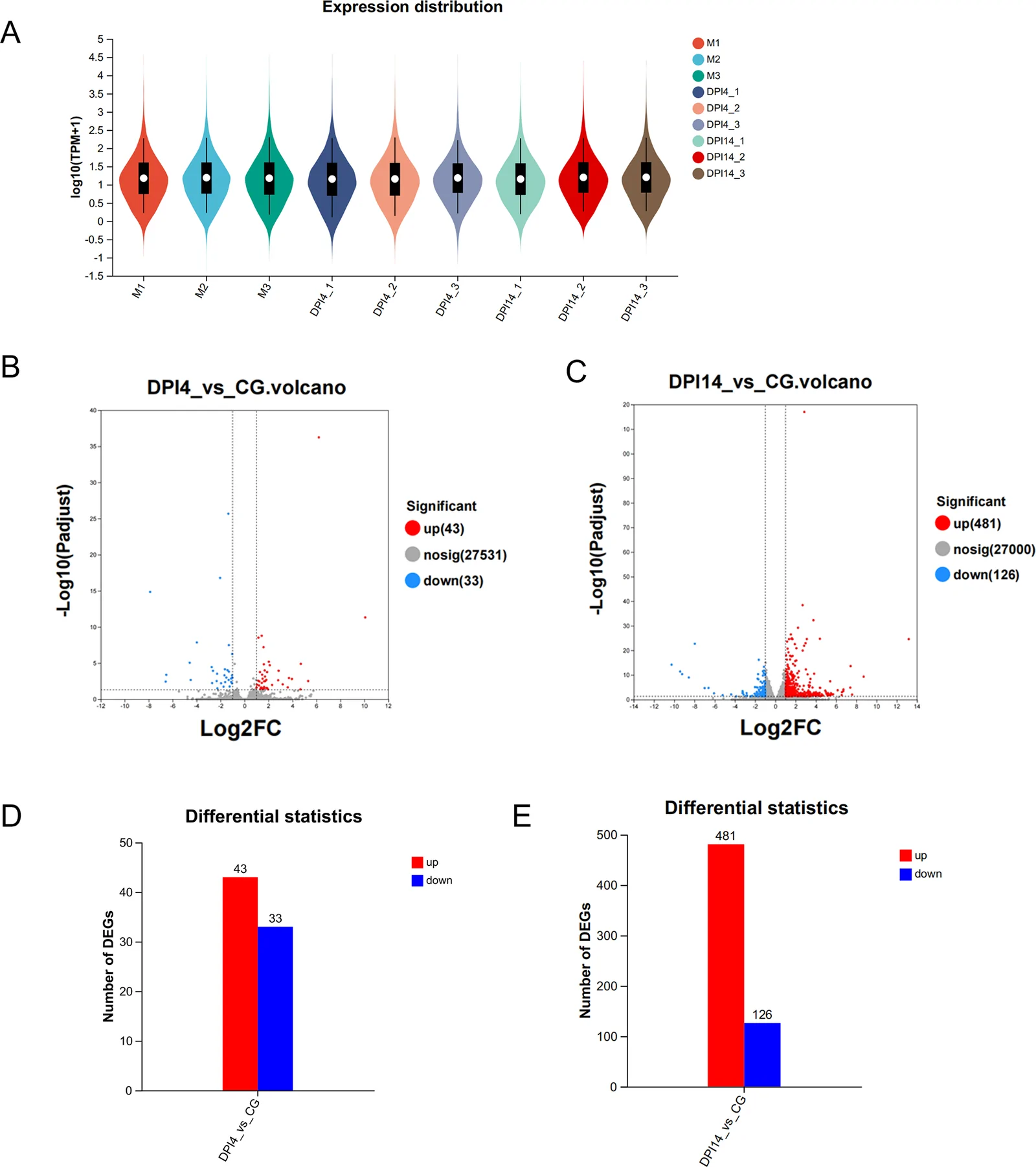
Time-resolved transcriptomic remodeling in TGs after BoHV-1 infection. TGs from control (CG), 4 dpi (DPI4), and 14 dpi (DPI14) calves (*n* = 3/group) were processed for mRNA-seq. (**A**) Violin plots of log_10_(TPM + 1) indicate comparable global expression distributions across groups. (**B and C**) Volcano plots for DPI4 vs CG and DPI14 vs CG with log_2_ fold-change and adjusted *P*-value thresholds indicated. (**D and E**) Numbers of DEGs at DPI4 and DPI14.

We performed mRNA-seq on TGs from control and infected calves at 4 and 14 dpi (DPI4 and DPI14; *n* = 3 per group). Total RNA was extracted using TRIzol. Poly(A)^+^ RNA was enriched with oligo-dT magnetic beads, converted to double-stranded cDNA, and libraries were sequenced using an Illumina NovaSeq 6000. Reads were quality-filtered (fastp) and aligned to the bovine reference genome (HISAT2/TopHat2). TPM-normalized expression distributions were comparable across samples, without apparent batch effects (Fig. 2A).

Given that viral DNA positivity in TGs at 4 dpi does not by itself establish active viral transcription and that low-abundance viral immediate-early transcripts may be underrepresented in whole-TG RNA-seq, we next performed targeted qRT-PCR analyses to determine whether early neuroinvasion was accompanied by immediate-early viral gene expression. To directly assess the presence of BoHV-1 immediate-early transcription in TGs during the early stages of infection, we performed qRT-PCR targeting the immediate-early genes bICP0 and bICP4. As expected, no viral transcripts were detected in the uninfected control TG. At 4 dpi, low but clearly detectable levels of bICP0 mRNA were observed (Fig. S5A). Since bICP0 transcripts can originate from either the IEtU1 promoter or the bICP0 early promoter, we additionally quantified the expression of bICP4, which is transcribed exclusively by the IEtU1 promoter. At 4 dpi, bICP4 transcripts were readily detectable in TGs but were absent in the control samples (Fig. S5B), confirming the presence of IEtU1-driven immediate-early viral transcription during early neuroinvasion. This indicates low-level viral transcriptional activity in TGs during early neuroinvasion, consistent with the early stages of infection. Although this analysis establishes that early TG infection is transcriptionally active, it does not by itself identify which intrinsic antiviral mechanisms regulate early viral progression in this context.

**Fig 3:**
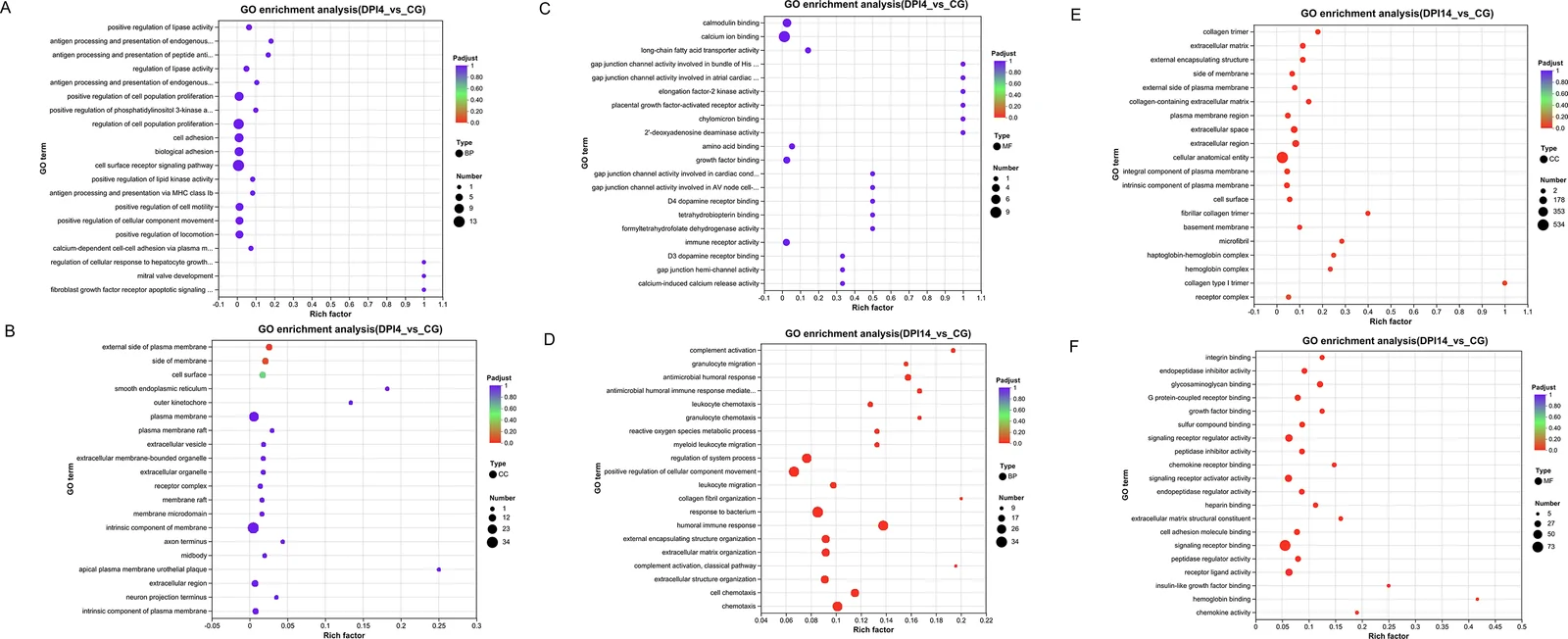
GO enrichment analysis of DEGs in TGs during BoHV-1 infection. TGs from control (CG), 4 dpi (DPI4), and 14 dpi (DPI14) calves (*n* = 3 per group) were subjected to mRNA-seq, read processing, mapping, and analysis. (**A–C**) GO enrichment analysis of DEGs at DPI4 reveals a relatively limited early transcriptional program dominated by pathways related to immune sensing, cell adhesion, and cell motility. (**D–F**) GO enrichment analysis of DEGs at DPI14 reveals broad activation of innate and humoral immune responses, accompanied by extracellular matrix remodeling and reduced neuronal/synaptic features. Dot size represents the number of genes associated with each GO term, and color indicates the Benjamini–Hochberg-adjusted *P*-value.

**Fig 4:**
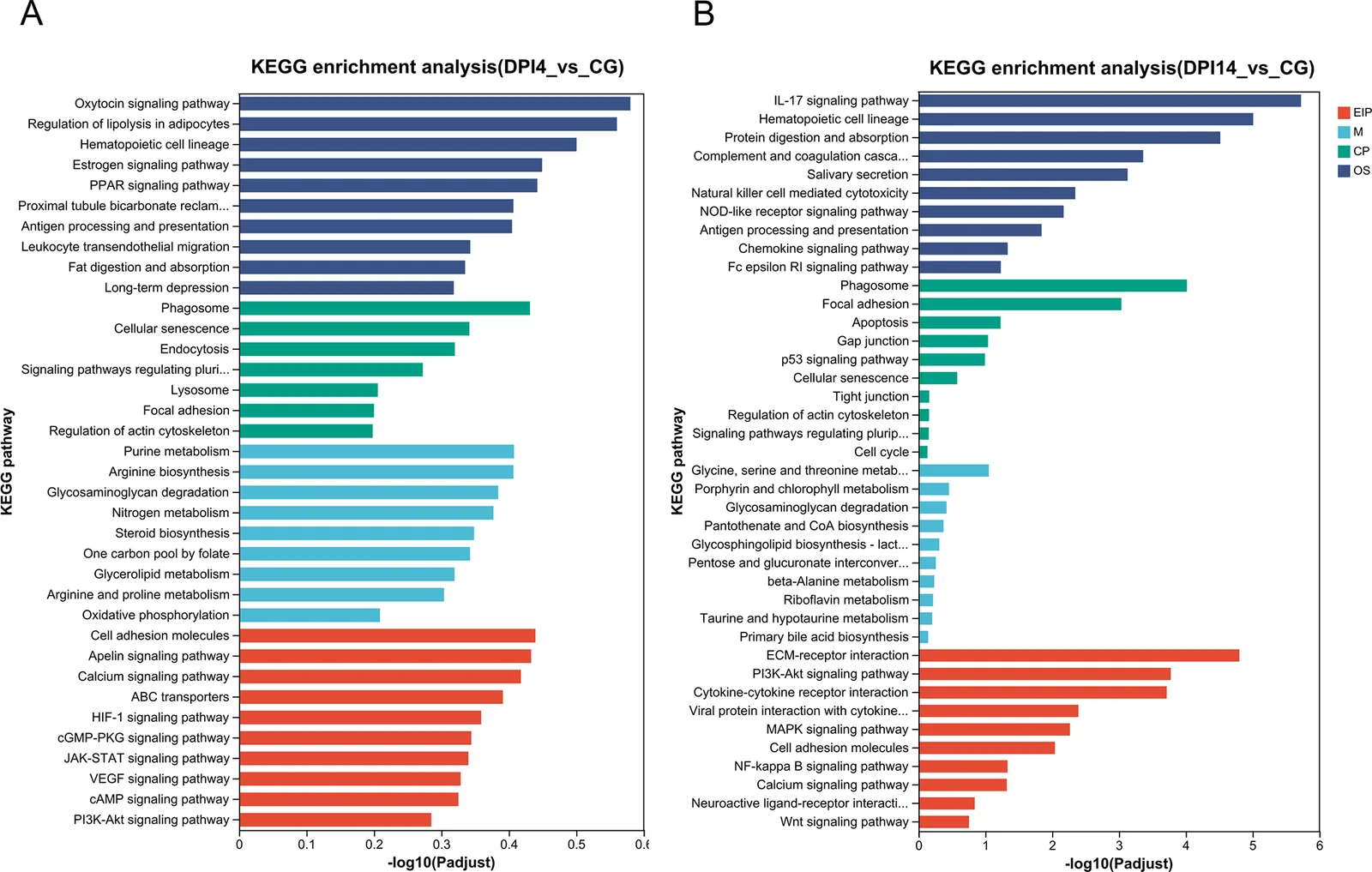
KEGG pathway enrichment analysis of differentially expressed genes in TGs during BoHV-1 infection. TGs from control (CG), 4 dpi (DPI4), and 14 dpi (DPI14) calves (*n* = 3 per group) were subjected to mRNA-seq, read processing, mapping, and analysis. (**A**) KEGG over-representation analysis of DEGs at DPI4 reveals enrichment of pathways related to endocytosis, intracellular trafficking, metabolic adaptation, and early signal priming. (**B**) KEGG over-representation analysis of DEGs at DPI14 reveals the dominance of the inflammatory and antiviral immune pathways, together with apoptosis- and extracellular-matrix-associated modules. Bars represent enriched KEGG pathways, ranked by −log_10_ Benjamini-Hochberg-adjusted *P*-value.

Relative to the control calves, modest transcriptional changes were detected at 4 dpi, with 76 DEGs identified (43 upregulated and 33 downregulated) (Fig. 2B and D). GO analysis revealed enrichment of antigen processing/presentation, cell adhesion/motility, and regulation of lipid-kinase-linked signaling (Fig. 3A); cellular component terms were enriched for the plasma membrane (including membrane rafts), receptor complexes, and extracellular vesicles (Fig. 3B); and molecular function terms were enriched for calcium/calmodulin binding and immune receptor activity (Fig. 3C). KEGG analysis suggested early engagement of the endocytosis/lysosome–phagosome pathways, leukocyte transendothelial migration, and signaling via JAK-STAT, PI3K–Akt, and second-messenger cascades (cAMP/cGMP/calcium) (Fig. 4A).

By 14 dpi, TGs exhibited broad transcriptional reprogramming, with 607 DEGs (481 upregulated and 126 downregulated) (Fig. 2C and E). The dominant GO biological process terms were complement activation, granulocyte chemotaxis, antimicrobial humoral responses, leukocyte chemotaxis, and reactive-oxygen-species metabolic processes (Fig. 3D); cellular component terms were enriched for ECM, collagen-containing ECM, and basement membrane (Fig. 3E); and the enriched molecular function terms included integrin binding, chemokine/cytokine receptor binding, and peptidase inhibitor/regulator activity (Fig. 3F). KEGG pathway analysis indicated robust activation of antiviral and inflammatory signaling pathways (IL-17, complement and coagulation cascades, NK cell-mediated cytotoxicity, NOD-/RIG-I-like receptor, chemokine, Toll-like receptor, NF-κB, MAPK, and TNF) together with apoptosis, p53, gap junction, and ECM–receptor interactions (Fig. 4B). Collectively, TGs exhibited early, limited priming at 4 dpi, followed by widespread innate/humoral immune activation and ECM remodeling at 14 dpi, consistent with neuroinvasion and a sustained antiviral/inflammatory milieu. The major biological processes and signaling pathways differing between the control and 4 dpi and between 4 and 14 dpi are summarized in Table S4.

### EggNOG/COG classification and GSEA reveal late-phase immune activation and synaptic downregulation in BoHV-1-infected TGs

**Fig 5:**
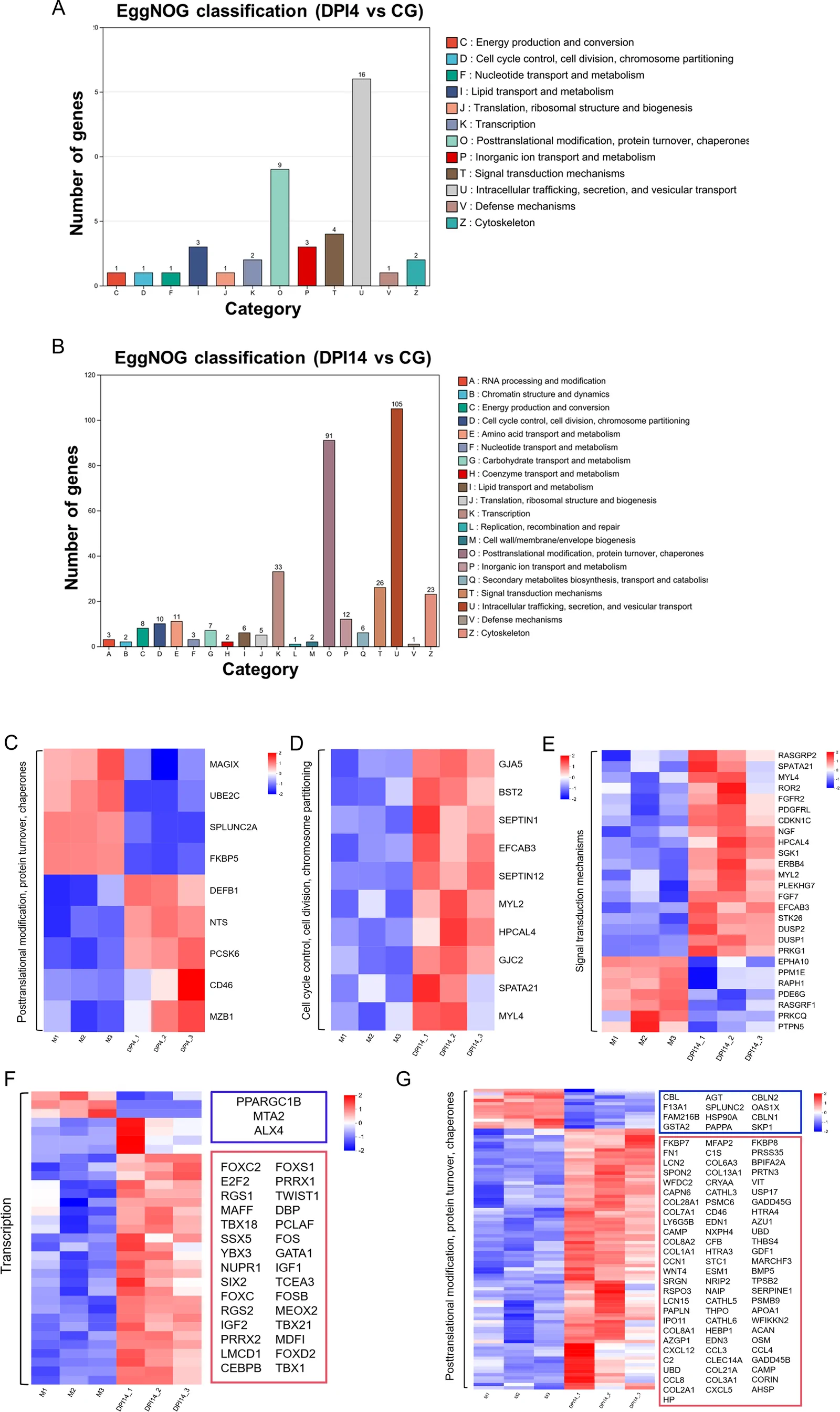
EggNOG/COG classification and representative gene modules in TGs after BoHV-1 infection. TG RNA-seq from control (CG), 4 dpi (DPI4), and 14 dpi (DPI14) calves (*n* = 3/group) was analyzed for differential expression and functional annotation. (**A and B**) DEGs (DPI4 vs CG; DPI14 vs CG) were categorized by EggNOG/COG. (**C–E**) Heat maps of selected transcription/signaling/metabolic modules showing progressive shifts from CG to DPI4 and DPI14. (**F and G**) Heat maps of immune and protein-turnover modules with dominant upregulation at DPI14. Color scale indicates *Z*-scored expression. Statistical thresholds are detailed in Materials and Methods.

**Fig 6:**
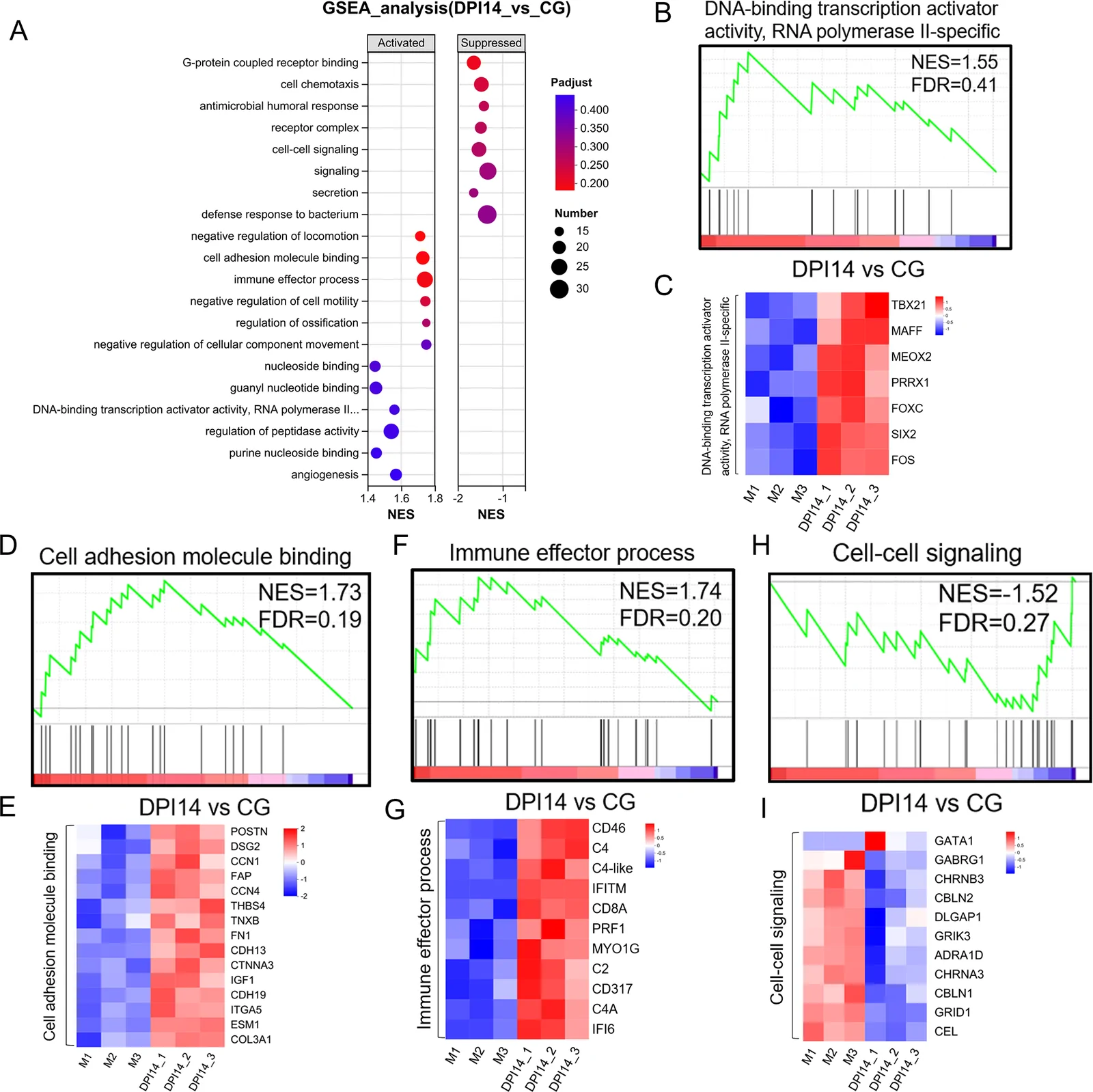
GSEA of TGs at 14 dpi versus controls. RNA-seq data from TGs (*n* = 3 per group) were analyzed via pre-ranked GSEA comparing expression at 14 dpi and in the control group, using a signed differential statistic to rank genes and MSigDB/GO collections as the gene sets, correcting for multiple testing. (**A**) Dot plot summarizing representative activated and suppressed gene sets with NES, FDR-adjusted *P*-values, and set sizes. (**B–G**) Enrichment plots with corresponding leading-edge heat maps for exemplar activated programs. (**H and I**) Enrichment plot and leading-edge heat map for a representative suppressed program. Heat maps of *Z*-scored expression across samples. Thresholds, databases, and permutation settings are detailed in Materials and Methods.

EggNOG/COG classification revealed a time-dependent pattern: at 4 dpi, the enrichment in K/O/T/I categories was consistent with early sensing and proteostasis priming (Fig. 5A and C), whereas at 14 dpi, enrichment was primarily in the V/T/O categories, together with D/L/U/Z, reflecting defense, signaling, PTM/turnover, cell cycle/repair, intracellular trafficking/secretion, and cytoskeleton (Fig. 5B, D through G). GSEA comparing the control and 14 dpi samples revealed positive enrichment of G-protein-coupled receptor binding, cell chemotaxis, antimicrobial humoral response, receptor complex, cell–cell signaling, and secretion (typical NES, ca. 1.55–1.74; FDR as shown; Fig. 6A through G). Negatively enriched sets included nucleoside/guanyl nucleotide binding, RNA polymerase II-related transcriptional activation, and regulation of peptidase activity (e.g., NES ca. −1.52; FDR ca. 0.27), with downregulation of synaptic receptor/neurotransmission modules (e.g., GABRG1, CHRNA3, GRID1, GRIK3, DLGAP1; Fig. 6H and I). Thus, at 14 dpi, TGs displayed immune/chemotactic activation with ECM remodeling, with suppression of neuronal/synaptic programs.

### Conserved PML–SUMO nuclear-body core with species-specific extensions identified in both human and bovine interactomes

Although KEGG and GO analyses identified multiple altered pathways in the TG, including lysosome/phagosome function, JAK–STAT signaling, and inflammatory response pathways, this does not directly reveal which intrinsic antiviral mechanisms may operate within infected neurons. Notably, the early (4 dpi) transcriptomic signature suggests enrichment in proteostasis-related functional categories (COG K/O/T/I), which are closely linked to SUMO- and ubiquitin-dependent regulation of nuclear protein complexes.

**Fig 7:**
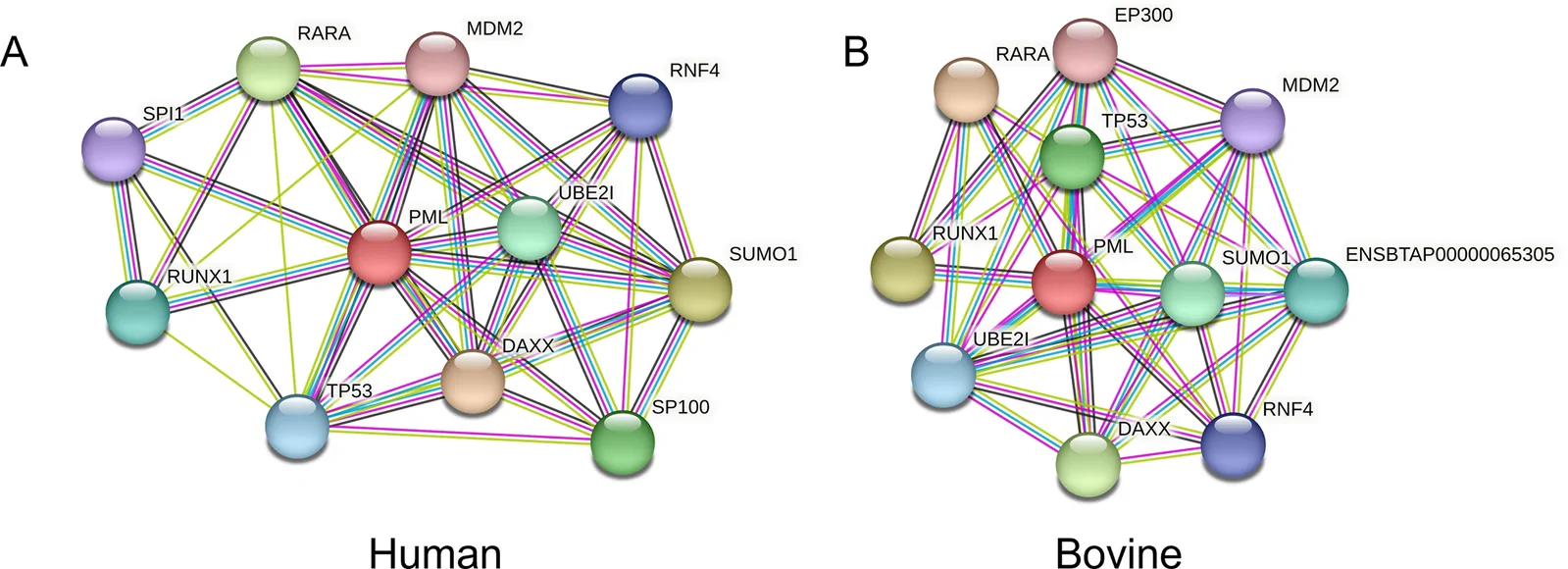
PML-centered protein–protein interaction networks in humans and cattle, generated using STRING. Networks were constructed in STRING using PML from *Homo sapiens* (**A**) and *Bos taurus* (**B**) as seed proteins, retrieving first-shell interactors under a high-confidence evidence model (based on evidence from curated databases, experiments, co-expression analysis, and text mining). Nodes represent proteins; edges depict STRING combined-score associations, and edge thickness reflects confidence. Layout, evidence channels, and enrichment procedures are described in Materials and Methods.

Given that PML-NBs represent a well-established intrinsic antiviral structure that integrates interferon responsiveness, proteostasis, and chromatin regulation, we used STRING analysis as a network-based approach to determine whether PML-centered interaction modules are represented within this context. This revealed densely interconnected PML-centered networks in the human and bovine data sets (Fig. 7A and B). For both species, PML is located at the hub of a canonical PML-NB module (PML–SUMO1–UBE2I–DAXX–SP100), indicating conservation of SUMO-dependent nuclear-body architecture. The networks converge on TP53–MDM2 and include RARA, RUNX1, and RNF4, thus linking PML to chromatin control, cell-cycle/stress signaling, and SUMO-triggered proteasomal turnover. Species-specific differences were evident; the human network contained SPI1 (PU.1), whereas the bovine network incorporated EP300 and an unannotated protein (ENSBTAP00000065305) connected to SUMO1/UBE2I/MDM2. These features suggest a conserved core with lineage-specific extensions that may tune the chromatin state and innate output.

### IFN-α–bPML axis suppresses very early replication via PML-NBs

**Fig 8:**
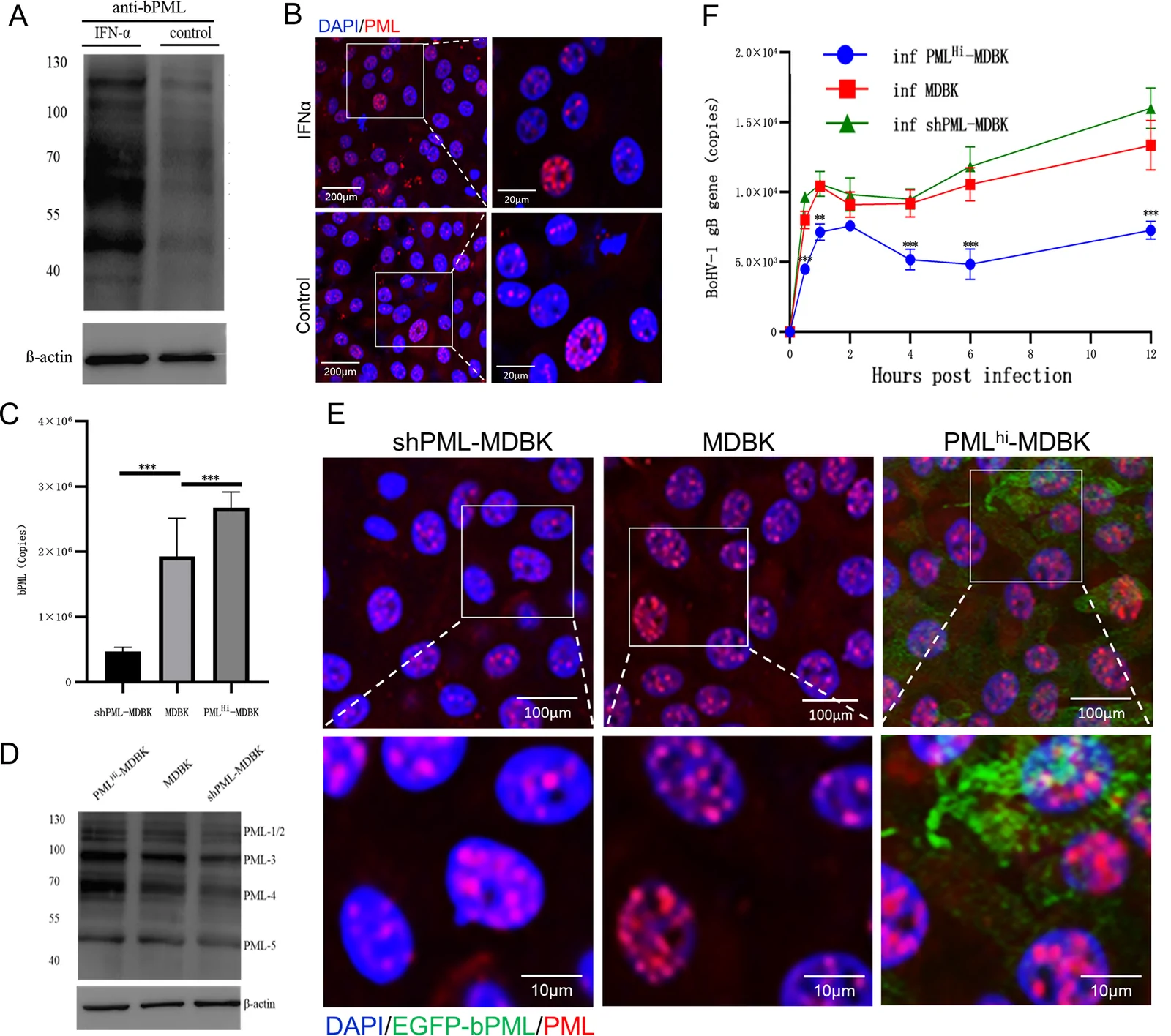
bPML expression modulates BoHV-1 infection and PML nuclear body organization in MDBK cells. (**A**) Immunoblotting of endogenous bPML protein in MDBK cells treated with IFN-α or left untreated (control), detected using an anti-bPML antibody, with β-actin as a loading control. (**B**) Immunofluorescence analysis of PML-NBs in MDBK cells following IFN-α treatment or in untreated controls. PML is shown in red, and nuclei are counterstained with DAPI (blue). Representative low- and high-magnification images are shown. (**C**) Quantification of BoHV-1 gB gene copies by qPCR in shPML-MDBK, wild-type MDBK, and PML^Hi^-MDBK cells at the indicated time point post-infection. (**D**) Immunoblotting of multiple bPML-immunoreactive protein species in shPML-MDBK, wild-type MDBK, and PML^Hi^-MDBK cells using an anti-bPML antibody, with β-actin as a loading control. Individual bands were not assigned to specific bPML isoforms. (**E**) Immunofluorescence of PML nuclear bodies in shPML-MDBK, wild-type MDBK, and PML^Hi^-MDBK cells. EGFP-bPML is shown in green, endogenous PML in red, and nuclei in blue (DAPI). Dashed boxes identify the regions shown below at higher magnification. (**F**) Time-course quantification of BoHV-1 gB gene copies by qPCR in infected shPML-MDBK, wild-type MDBK, and PML^Hi^-MDBK cells. Immunoblot bands were not assigned to specific isoforms and represent total bPML-immunoreactive species. Data are shown as mean ± SD. Statistical analyses and the number of biological replicates are described in Materials and Methods. ns, not significant; *P < 0.05; **P < 0.01; ***P < 0.001; ****P < 0.0001.

To connect these *in vivo* observations to a tractable antiviral mechanism, we examined the PML-centered intrinsic defense axis in cell culture. This step was motivated by the TG transcriptomic signatures suggesting IFN- and proteostasis-related host responses, together with the established role of PML as an interferon-responsive intrinsic antiviral factor. Given that PML participates in IFN-α-mediated innate immunity (7), we examined whether IFN-α regulates bPML in MDBK cells. IFN-α stimulation markedly increased bPML protein levels (Western blot; Fig. 8A), and immunofluorescence revealed pronounced remodeling of PML-NBs, with clear increases in both number and size (Fig. 8B). To assess their function, we generated shPML-MDBK (knockdown) and PML^Hi^-MDBK (overexpression) derivatives. qPCR/Western blotting confirmed the expected bPML differences, and immunofluorescence revealed corresponding changes in PML-NBs (Fig. 8C through E). Following infection at MOI = 1, quantification of viral gB copies at 0.5–12 hpi revealed that PML^Hi^-MDBK cells accumulated significantly less viral DNA than the parental MDBK and shPML-MDBK cells (*P* < 0.05), whereas the shPML-MDBK cells tended to harbor more viral DNA than the parental MDBK cells (although this difference was not significant) (Fig. 8F). Thus, IFN-α enhances bPML abundance and NB organization, and elevated bPML constrains very early BoHV-1 replication.

### BoHV-1 subverts bPML restriction by dismantling PML-NBs

**Fig 9:**
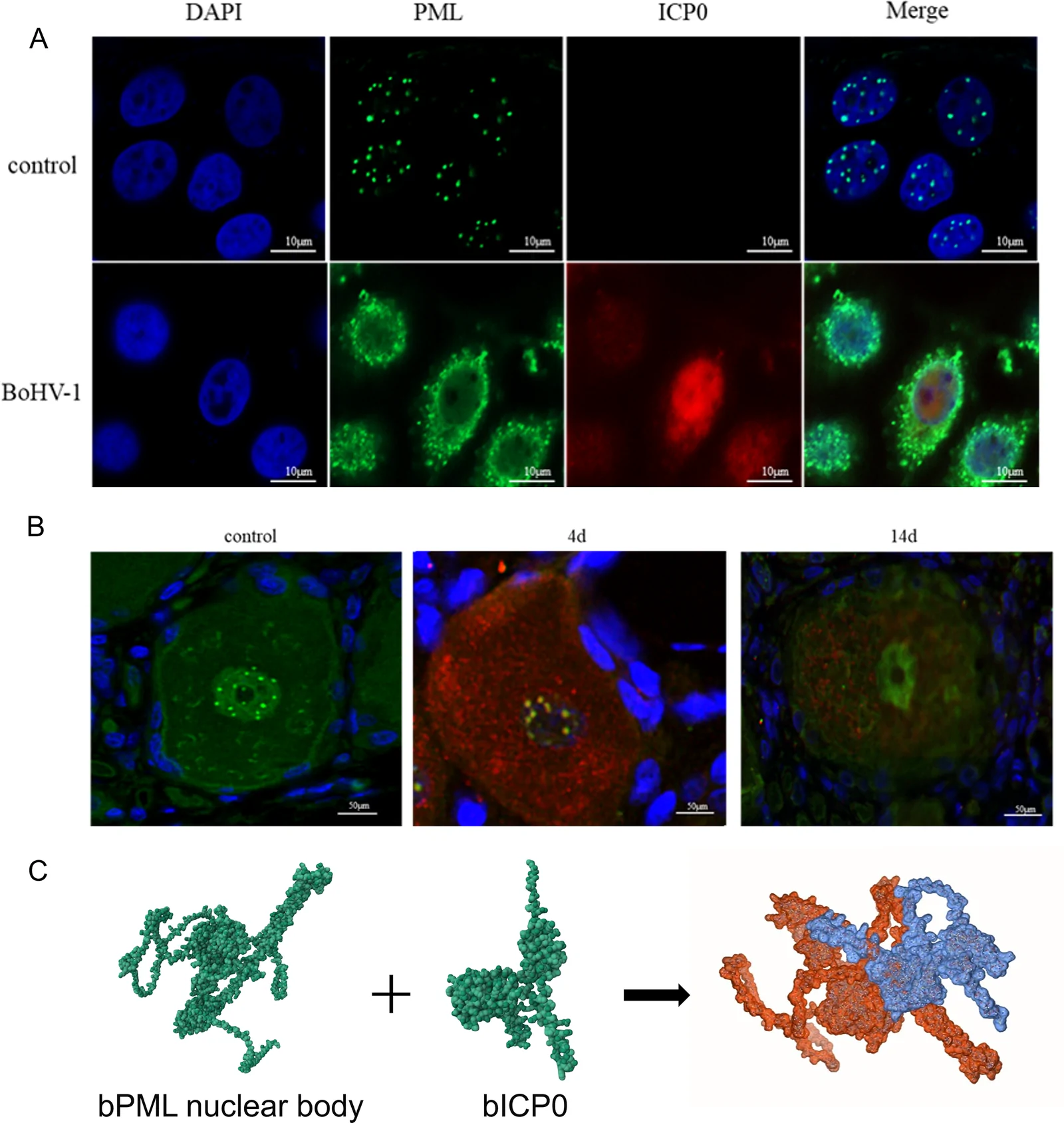
BoHV-1 targets PML nuclear bodies *in vitro* and *in vivo*, with a plausible bPML–bICP0 interface supported by docking. (**A**) MDBK cells infected with BoHV-1 (MOI = 1) were fixed at the onset of CPE and stained with anti-bPML (green), anti-ICP0 (red), and DAPI (blue); scale bars as indicated. (**B**) TGs from infected calves were collected at 4 and 14 dpi, sectioned, and subjected to double immunofluorescence with anti-bPML (green) and anti-ICP0 (red) plus DAPI counterstaining (blue); scale bars as indicated. (**C**) Protein–protein docking: the bPML–bICP0 interaction was modeled using HADDOCK; the top cluster (*n* = 30) yielded a score of −53.0 ± 3.5 and a buried surface area of 994 ± 67 Å². Docking parameters and evaluation criteria are detailed in Materials and Methods.

To test whether BoHV-1 attenuates bPML-mediated restriction by targeting PML-NBs, we generated and validated bPML-specific reagents. Recombinant bPML produced in *Escherichia coli* (pET-32a-bPML/Transetta[DE3]) migrated at ca. 55 kDa, based on SDS–PAGE; Ni-NTA chromatography yielded a single predominant species, and immunoblotting confirmed its identity with anti-His and anti-bPML antibodies (Fig. S2A through D). Using the purified antigen, we raised rabbit/mouse polyclonals and obtained a monoclonal antibody (bPML-2G5) via hybridoma screening; bPML-2G5 specifically recognized exogenous bPML in 293T cells, based on immunofluorescence and immunoblotting (Fig. S3A through C). Immunofluorescence of BoHV-1-infected MDBK cells at the onset of CPE revealed loss of discrete nuclear PML-NB puncta, fragmentation/dispersion of NB structures, and partial relocalization of bPML to the cytoplasm (Fig. 9A). *In vivo*, double immunofluorescence of trigeminal ganglion sections qualitatively revealed nuclear colocalization of bPML with ICP0 at 4 dpi, whereas at 14 dpi, NB architecture was disorganized, with fewer and irregular puncta and a more diffuse nuclear signal (Fig. 9B). Complementary protein–protein docking supported a direct bPML–bICP0 interface (top HADDOCK cluster score, −53.0 ± 3.5; Fig. 9C). Together, these results reveal bidirectional antagonism: intact PML-NBs contribute to early restriction of BoHV-1 by bPML, and the virus counters this by dismantling PML-NB integrity.

### Isoform-specific modulation of early BoHV-1 replication by bPML

**Fig 10:**
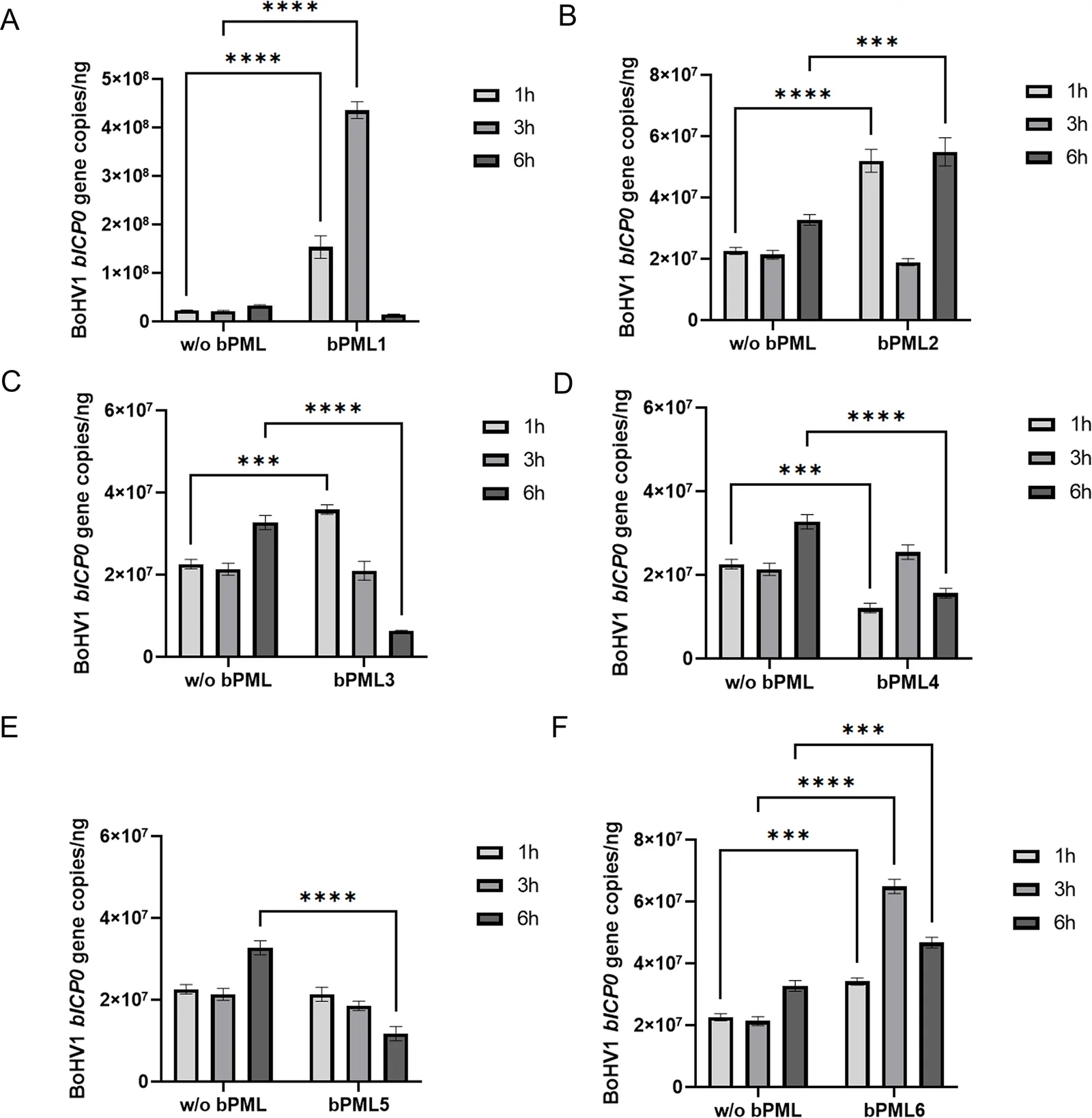
bPML isoforms differentially modulate early BoHV-1 bICP0 gene accumulation. Vero cells transiently expressing individual bPML isoforms (bPML1–bPML6) or a vector control (w/o bPML) were infected with BoHV-1 (MOI = 1). (**A–F**) At 1, 3, and 6 hpi, qPCR was used to quantify bICP0 gene copies (mean ± SD; significance indicated in the plots). Statistical tests and replication details are provided in the Materials and Methods section. ns, not significant; *P < 0.05; **P < 0.01; ***P < 0.001; ****P < 0.0001.

**Fig 11:**
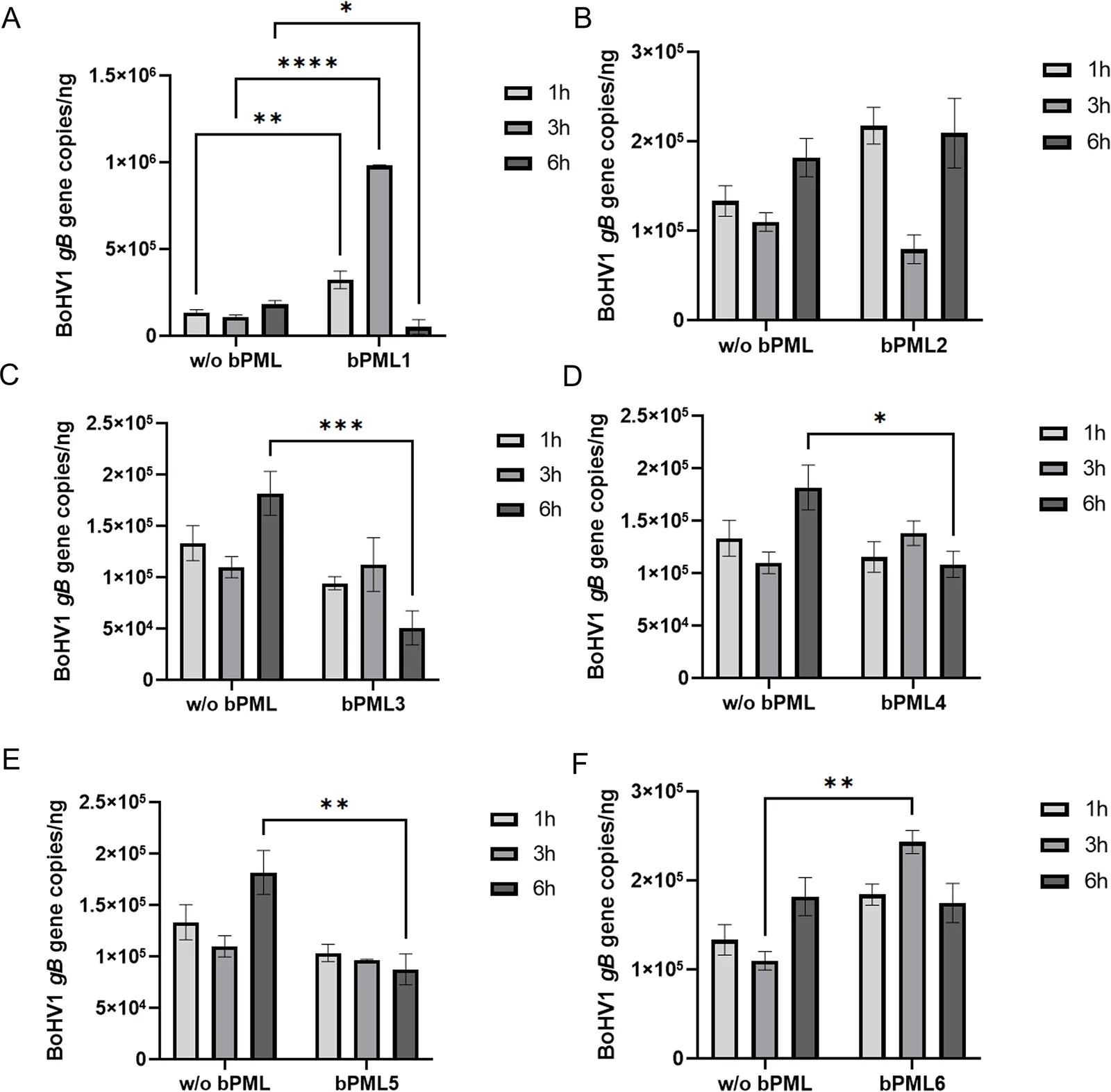
bPML isoforms differentially modulate early BoHV-1 gB gene accumulation. Vero cells transiently expressing individual bPML isoforms (bPML1–bPML6) or a vector control (without bPML) were infected with BoHV-1 (MOI = 1). (**A–F**) At 1, 3, and 6 hpi, qPCR was used to quantify gB gene copies (mean ± SD; significance indicated in the plots). Statistical tests and replication details are provided in the Materials and Methods section. ns, not significant; *P < 0.05; **P < 0.01; ***P < 0.001; ****P < 0.0001.

To determine isoform specificity, Vero cells were transiently transfected with bPML1–bPML6 and infected (MOI = 1). Viral immediate-early (bICP0) and structural (gB) gene copies were quantified at 1, 3, and 6 hpi. The isoforms exhibited clear bidirectional effects: transfection with bPML1 and bPML6 resulted in consistently higher bICP0/gB copy numbers (strongest effect at 3–6 hpi; Fig. 10A and F and 11A and F), while transfection with bPML2 produced a modest increase (Fig. 10B and 11B). Transfection with bPML3, bPML4, and bPML5 reduced bICP0/gB copy accumulation over the time course, with bPML5 achieving the most stable suppression over the time course (Fig. 10C through E , and 11C through E). The differences were minimal at 1 hpi, becoming pronounced by 3–6 hpi, indicating isoform-specific, opposing control over very early genome accumulation.

## DISCUSSION

In this study, we integrated time-resolved *in vivo* analysis of BoHV-1 infection in calves with targeted *in vitro* experiments designed to examine the intrinsic antiviral mechanisms suggested by analysis of the TG transcriptome. Although the calf model defines the timing and tissue environment of mucosal replication and trigeminal ganglion neuroinvasion, it does not allow direct mechanistic testing of candidate host antiviral pathways. Therefore, we employed reductionist cell culture systems to experimentally test whether a PML-centered intrinsic antiviral axis could influence very early viral infection and be targeted by BoHV-1. Importantly, these *in vitro* experiments were designed as a mechanistic extension of the *in vivo* findings, rather than as an independent descriptive component.

Our controlled calf infection model delineates a coherent sequence in BoHV-1 pathogenesis. Viral shedding was predominantly from the upper-airway mucosa: titers were highest in the nasal mucosa, lower in the ocular mucosa, and substantially lower in rectal mucosa. Viral titers peaked ca. 3–6 dpi and contracted by 10–14 dpi; at 4 dpi, the virus was distributed primarily in the tonsils and URT, with a lower yet detectable signal in TGs; at 14 dpi, TGs remained positive for the virus, despite the falling mucosal load, indicating neuroinvasion and early viral DNA persistence in TGs during the late acute phase (2, 15, 19). Transcriptomically, TGs exhibited a two-phase program, with limited sensing/proteostasis priming at 4 dpi (COG K/O/T/I) and broad immune–ECM activation at 14 dpi (COG V/O/T with D/L/U/Z), with GSEA revealing enrichment of chemotaxis and complement/humoral modules and repression of synaptic programs, mirroring tissue virologic kinetics (4, 19, 20).

Host proteostasis is a unifying mechanism for both antiviral restriction and counteraction (21, 22). EggNOG/COG classification at 14 dpi revealed post-translational modification/protein turnover/chaperones, and GSEA revealed coordinated upregulation of ISG/antigen processing and proteasome modules. Because PML-NB integrity requires SUMOylation, poly SUMOylated PML is recognized by SUMO-targeted ubiquitin ligases (e.g., RNF4) for proteasomal degradation, and enhanced PTM/turnover networks can simultaneously amplify antiviral proteostasis within NBs and create an avenue for bICP0-driven NB disassembly via SUMO–ubiquitin crosstalk (23–25). Consistent with prior reports demonstrating the nuclear-trafficking capacity of BoHV-1 tegument proteins (26), our findings provide evidence that viral nuclear components engage the host SUMOylation machinery and PML-NBs to promote productive infection. The results of cross-species STRING mapping were concordant with this model, revealing a conserved PML–SUMO1–UBE2I–DAXX–SP100 core linked to TP53–MDM2 and RNF4 in both human and bovine networks, with species-specific extensions (e.g., SPI1 in humans; EP300 and a bovine SUMO/ubiquitin-linked node in cattle) that may tune chromatin states and innate outputs (27–29).

Mechanistically, in cell-culture systems, the IFN-α–PML axis constrains very early replication, and BoHV-1 subsequently counteracts this defense (30–32). IFN-α upregulated bPML and enlarged PML-NBs, and bPML elevation reduced BoHV-1 genome accumulation at 0.5–12 hpi, whereas bPML knockdown tended to increase it, supporting PML as an intrinsic, IFN-sensitized antiviral hub (11, 17). Conversely, BoHV-1 compromised PML-NB integrity: MDBK cells at CPE onset exhibited fragmentation/dispersion of nuclear PML puncta and partial cytoplasmic relocalization; in TG, nuclear bPML/ICP0 colocalization at 4 dpi led to disorganized NB architecture at 14 dpi. Docking analysis supported a direct, energetically favorable bPML–bICP0 interface. This bidirectional antagonism is consistent with established HSV-1 ICP0–PML interactions, in which ICP0 targets PML-NBs for degradation and disrupts ND10-mediated intrinsic antiviral restriction (8, 9). Recent studies have indicated that ICP0 exhibits isoform-specific targeting of PML, suggesting that the differential recognition of PML isoforms contributes to the selective modulation of PML-NB-mediated antiviral restriction (8, 33). In this context, ICP0-mediated targeting of PML and associated components, such as SP100, leads to the dismantling of PML-NBs and relieves the intrinsic restriction, supporting the interpretation that bICP0 executes a functionally analogous countermeasure in bovine cells (11, 34).

Our isoform analysis results suggest that PML’s antiviral output is modular and tunable (13, 35). In Vero cells, bPML1/6 (and to a lesser extent, bPML2) enhanced early bICP0/gB accumulation, whereas bPML3/4/5 suppressed it, with bPML5 achieving the most stable inhibition. Such isoform dependence, which has been reported for human PML in HSV models (36, 37), likely reflects differences in the C-terminal SUMO-interacting motifs, partner recruitment (e.g., DAXX/SP100), and NB ultrastructure. Our findings extend this principle to bovine PML and BoHV-1, implying that isoforms bias PML-NB composition toward restriction-competent or restriction-deficient states.

These molecular events have clear implications for the TG niche (38, 39). By 14 dpi, the microenvironment displayed chemokine-rich ECM-remodeling signatures and suppression of neuronal/synaptic programs, establishing an antiviral glial–neuronal milieu that prioritizes leukocyte recruitment and antigen processing over neurotransmission, thus potentially constraining viral spread at the cost of transient neuronal function. Given the role of PML at the intersection of the IFN signaling, chromatin regulation, and stress pathways (7, 29, 40), the PML-NB state may influence how TGs balance immune activation with neuronal homeostasis in the late acute phase.

These findings are limited by the modest group sizes (*n* = 3), which limits the power to detect subtle effects and precludes stratification by cell type; by the reliance on Vero/MDBK systems, which capture conserved mechanisms but lack TG cellular diversity; and by the focus on the 4 and 14 dpi time points. Furthermore, as the calves received colostrum from non-vaccinated dams, BoHV-1 infection occurred in the presence of maternally derived antibodies, which may have partially modulated early viral replication and immune responses. Examination of effects at intervening or later intervals (e.g., 21–35 dpi) and in bona fide reactivation models are needed to map the continuum into latency and reactivation (2, 41). Consistent with established BoHV-1 latency models, true latency in TGs is typically defined at later time points (at 28–35 dpi or ≥60 dpi), and incorporating these windows is a key objective for our future *in vivo* studies (41, 42). Viral DNA copies were used as readouts, and integrating infectious titers and immediate-early and late transcripts would refine replication staging. Finally, while STRING-based PPI networks can be used to infer functional proximity, they do not establish direct physical binding for all edges; thus, targeted biochemical validation is essential.

We are currently planning follow-up studies to quantitatively assess PML-NB disruption *in vivo* during the later stages of infection or reactivation, when a greater number of infected neurons can be examined. In our future work, we will examine whether the RING and SIM-interacting surfaces of bICP0 are necessary or sufficient for dismantling NBs; define isoform-specific bPML domains that govern restriction and bICP0 sensitivity; perturb SUMO/ubiquitin enzymes (UBE2I, PIAS, and RNF4) while monitoring NB dynamics and BoHV-1 output; deploy ChIP-seq/ATAC-seq and proximity proteomics for TGs, to connect PML-NB states to chromatin and partner composition; and apply single-cell/spatial transcriptomics to assign TG programs to neurons, satellite glia, and infiltrating leukocytes. Translationally, leveraging IFN-α or small-molecule enhancers of PML-NB integrity, while avoiding imbalances in proteostasis that favor NB dismantling, may reduce acute replication and limit seeding of latency, although this requires *in vivo* validation of efficacy and safety in cattle.

## Data Availability

The RNA-seq data generated in this study have been deposited in the NCBI under BioProject accession number PRJNA1073724. Raw sequencing data are available in the Sequence Read Archive (SRA) under run accessions SRR27884958–SRR27884966.

## SUPPLEMENTAL MATERIAL

Supplemental figures spectrum.02698-25-s0001.docx Fig. S1 to S5.

Supplemental tables spectrum.02698-25-s0002.docx Tables S1 to S5.

ASM does not own the copyrights to Supplemental Material that may be linked to, or accessed through, an article. The authors have granted ASM a non-exclusive, world-wide license to publish the Supplemental Material files. Please contact the corresponding author directly for reuse.
