# Supplementary material for: Time-resolved mapping in calves reveals bovine herpesvirus 1 shift from mucosal replication to trigeminal ganglion neuroinvasion with promyelocytic leukemia protein-centered host–virus antagonism
Source: Microbiol Spectr. 2026 Jul 23;14(9):e02698-25. doi: 10.1128/spectrum.02698-25 (PMC13532201; doi:10.1128/spectrum.02698-25)
Supplement: Supplementary file 1 [file spectrum.02698-25-s0001.docx]

**Supplementary Figures**

**Supplementary Figure S1. qPCR-based kinetics of BoHV-1 shedding in experimentally infected calves.**
Calves were inoculated intranasally and ocularly with BoHV-1 (1.4 × 10^6 pfu per head). From 1 to 14 days post-infection (dpi), (A)ocular, (B)nasal, and (C)rectal swabs were collected daily. Viral DNA was extracted and quantified by absolute qPCR targeting the gB gene using a plasmid standard curve (details in Methods). Curves/bars depict BoHV-1 genome copies per swab for individual calves with group mean ± SD (n = 3).

**Supplementary Figure S2: Expression and purification of recombinant bPML.**

(A–C) SDS–PAGE analysis of bPML expression, solubility, and Ni-NTA purification from Transetta (DE3) harboring pET-32a-bPML. (D) Western blot confirming the ~55 kDa recombinant bPML band in the purified fraction (detected with anti-His or anti-bPML, as indicated).
**Lane assignments:** M, protein ladder; 1, pET-32a(+) empty-vector control; 2, non-induced culture; 3–4, induced cultures (IPTG, 25 °C, 7 h); 5, soluble (supernatant) fraction of induced lysate; 6, insoluble (pellet) fraction; 7, unpurified recombinant bPML; 8, Ni-NTA-purified recombinant bPML.

C

**Supplementary Figure S3: Preparation of the antibodies against bovine PML**

(A-B) IFA: The pCMV-HA-C-bPML plasmid, which contains the full-length bPML gene, was transfected into 293T cells. Subsequently, were treated with fixation and permeabilization, followed by incubation with mAb bPML-2G5 as the primary antibody.

(C) Western blot: Whole cell lysates from 293T cells exogenously transfected with the pCMV-HA-C-bPML plasmid were analyzed by immunoblotting using mAb bPML-2G5.

**Supplementary Figure S4: Early tissue distribution of BoHV-1 DNA in experimentally infected calves and uninfected controls.**
Calves were inoculated intranasally and ocularly with BoHV-1 (1.4 × 10⁶ PFU per head) and euthanized at 4 days post-infection (dpi) or uninfected controls. Palatine tonsil, trigeminal ganglion (TG), and additional upper-airway and lymphoid tissues were collected. Total DNA was extracted, and viral genomes were quantified by absolute qPCR targeting the gB gene. Bars show gB copy numbers normalized to input tissue DNA for each animal; horizontal lines denote group mean ± SD (n = 3 calves per time point). Statistical methods and significance criteria are described in the Methods section. ND, not detected by qPCR (Ct > 40 for all replicates).

B

A

**Supplementary Figure S5: BoHV-1 immediate-early transcripts in TG at 4 dpi.** Quantitative RT-PCR detection of the BoHV-1 immediate-early gene (A) bICP0 and (B) bICP4 in trigeminal ganglia (TG). Each point represents one calf (sampled at 4 dpi or control). Bars indicate mean ± SD. The red dashed line denotes the limit of detection (LOD). Y-axis represents bICP0 copies per μg RNA (log₁₀ scale).
