# Supplementary material for: Time-resolved mapping in calves reveals bovine herpesvirus 1 shift from mucosal replication to trigeminal ganglion neuroinvasion with promyelocytic leukemia protein-centered host–virus antagonism
Source: Microbiol Spectr. 2026 Jul 23;14(9):e02698-25. doi: 10.1128/spectrum.02698-25 (PMC13532201; doi:10.1128/spectrum.02698-25)
Supplement: Supplementary file 2 [file spectrum.02698-25-s0002.docx]

# Supplementary Materials

This file contains detailed information on primer sequences, oligonucleotides and antibodies used in this study. Data are presented in tabular format as required for reproducibility.

## Table S1. Primer sequences used for qPCR and RT-qPCR

| **Target** | **Forward (5′→3′)** | **Reverse (5′→3′)** |
| --- | --- | --- |
| BoHV-1 gB | GGAACTACACGGAGGGCATC | CGGTCCGTGTACTGGTTTGT |
| BoHV-1 bICP0 | TGGTGTTTCTCGACACCTCG | TCCGTTTCCGTGTCGTCAAT |
| BoHV-1 bICP4 | GCCACAGCTCGTTCATCAC | GCTTCTGGTCGCAGTCGTAG |
| GAPDH | TGCACCACCAACTGCTTAGC | GGCATGGACTGTGGTCATGA |

## Table S2. shRNA oligonucleotides targeting bovine PML

| shRNA ID | Sense (5′→3′) | Antisense (5′→3′) |
| --- | --- | --- |
| shPML-1953 | CCGGGCACTTCCTGCATTTCCTAGCCTCGAGGCTAGGAAATGCAGGAAGTGCTTTTTG | AATTCAAAAAGCACTTCCTGCATTTCC TAGCCTCGAGGCTAGGAAATGCAGGAAGTGC |
| shPML-2041 | CCGGGCTCTGGAGGAGATGAACATGCTCGAGCATGTTCATCTCCTCCAGAGCTTTTTG | AATTCAAAAAGCTCTGGAGGAGATGAACATGCTCGAGCATGTTCATCTCCTCCAGAGC |
| shPML-2429 | CCGGGCCTGAAGAAGTACAACCGATCTCGAGATCGGTTGTACTTCTTCAGGCTTTTTG | AATTCAAAAAGCCTGAAGAAGTACAACCGATCTCGAGATCGGTTGTACTTCTTCAGGC |
| shPML-2496 | CCGGGCATTCTCTAGGCACCTACTTCTCGAGAAGTAGGTGCCTAGAGAATGCTTTTTG | AATTCAAAAAGCATTCTCTAGGCACCTACTTCTCGAGAAGTAGGTGCCTAGAGAATGC |

**Table S3. Antibodies used in this study**

| **Antibody / Reagent** | **Description** |
| --- | --- |
| Rabbit polyclonal anti-bPML | Generated against recombinant bPML antigen; validated by WB and IF |
| Mouse polyclonal anti-bPML | Raised against purified recombinant bPML |
| Mouse monoclonal anti-bPML (clone bPML-2G5) | Hybridoma-derived; validated by WB and IF |
| Anti-His antibody | Abcam, Cambridge, MA, USA |
| Secondary antibodies | Cell Signaling Technology, Danvers, MA, USA |
| DAPI | Invitrogen, Thermo Fisher Scientific, Carlsbad, CA, USA |

**Table S4. Summary of major biological processes and signaling pathways altered in TG after BoHV-1 infection**

| **Comparison** | **Representative enriched biological processes (GO, BP)** |
| --- | --- |
| **Control → 4 dpi** | • Antigen processing and presentation of peptide antigen  • Regulation of lipid-kinase signaling  • Cell adhesion and leukocyte migration  • Calcium ion binding and receptor complex organization  • Membrane raft and extracellular vesicle formation |
| **4 dpi → 14 dpi** | • Complement activation and humoral immune response  • Chemokine and leukocyte chemotaxis  • Antimicrobial peptide production  • Reactive oxygen species and oxidative stress metabolism  • Extracellular matrix organization, collagen fibril formation, and integrin binding |

| **Comparison** | **Key signaling pathways (KEGG)** |
| --- | --- |
| **Control → 4 dpi** | • Endocytosis / Phagosome / Lysosome  • Leukocyte transendothelial migration  • JAK–STAT, PI3K–Akt, and cAMP/cGMP/calcium signaling  • Apelin and VEGF signaling  • Fat digestion and absorption |
| **4 dpi → 14 dpi** | • IL-17, NF-κB, TNF, and MAPK signaling  • Toll-like, NOD-like, and RIG-I-like receptor pathways  • Cytokine–cytokine receptor interaction  • Complement and coagulation cascades  • p53, apoptosis, ECM–receptor interaction, gap junction |

**Table S5. NCBI sequence accession numbers corresponding to the bovine PML isoforms cloned in this study.**

| bPML-I: | NM_001206503.1 |
| --- | --- |
| bPML-II: | XM_005221906.5 |
| bPML-III: | XM_005221911.5 |
| bPML-IV: | XM_005221912.5 |
| bPML-V: | XM_010817047.4 |
| bPML-VI: | OM_324027.1 |
